# Closing the diagnostic gap in male genital schistosomiasis (MGS): current detection tools and novel strategies

**DOI:** 10.1017/S0031182025000599

**Published:** 2025-12

**Authors:** Bronwyn Neufeld, Sergio España-Cueto, Lisette van Lieshout, Bonnie L. Webster, Pytsje T. Hoekstra, Sekeleghe Kayuni, J. Russell Stothard, Tegwen Marlais, Shinjiro Hamano, Amaya L. Bustinduy

**Affiliations:** 1 Department of Clinical Research, London School of Hygiene & Tropical Medicine, London, UK; 2 School of Tropical Medicine and Global Health, Nagasaki University, Nagasaki, Japan; 3 Department of Infectious Diseases, Hospital Universitari Germans Trias i Pujol. Fundació Lluita contra les Infeccions, Badalona, Spain; 4 University of Vic–Central University of Catalonia (UVic-UCC), Vic, Spain; 5 Leiden University Center for Infectious Diseases, Subdepartment Research (LUCID-R); Leiden University Medical Center, Leiden, the Netherlands; 6 Natural History Museum, London, UK; 7 Malawi-Liverpool-Wellcome Programme, Kamuzu University of Health Sciences, Queen Elizabeth Central Hospital Campus, Blantyre, Malawi; 8 Department of Tropical Disease Biology, Liverpool School of Tropical Medicine, Liverpool, UK; 9 Department of Infection Biology, London School of Hygiene & Tropical Medicine, London, UK; 10 Department of Parasitology, Institute of Tropical Medicine (NEKKEN), Nagasaki University, Nagasaki, Japan

**Keywords:** diagnostics, male genital schistosomiasis, *Schistosoma haematobium*, semen

## Abstract

Male genital schistosomiasis (MGS), a gender-specific manifestation of urogenital schistosomiasis and neglected tropical disease, typically results from the entrapment of *Schistosoma haematobium* eggs within the male genital tract. Across the world, there are no current and accurate estimates of the burden of MGS, due to disease underreporting primarily from diagnostic challenges and a lack of general awareness within the health system. Diagnostic methods for MGS are extremely limited. Conventionally, semen microscopy for *Schistosoma* ova is used though this technique suffers from low sensitivity and lacks protocol standardization. The introduction of molecular diagnostics, such as polymerase chain reaction (PCR), has partly helped overcome this challenge of low sensitivity, though may not be suitable for use in resource-constrained settings. To address these challenges, in this review, we propose a two-step diagnostic algorithm for MGS in accordance with recent WHO guidelines, consisting of a high sensitivity serological test followed by a high specificity test (microscopy or molecular assay, dependent on setting). Further investigation is required into standardization of sample collection, processing, storage, and analysis in order to identify an evidence-based optimal diagnostic pipeline. New diagnostic tools are needed such as isothermal molecular assays, alongside optimization for semen analysis, which may alleviate barriers to diagnosis and present opportunities for integration with other sexual and reproductive health screening. These areas of future investigation underpin the development of a suitable diagnostic pipeline, as the continued neglect of MGS and its underdiagnosis presents a threat to the goal of elimination of schistosomiasis as a public health problem.

## Graphical abstract

**Figure:**
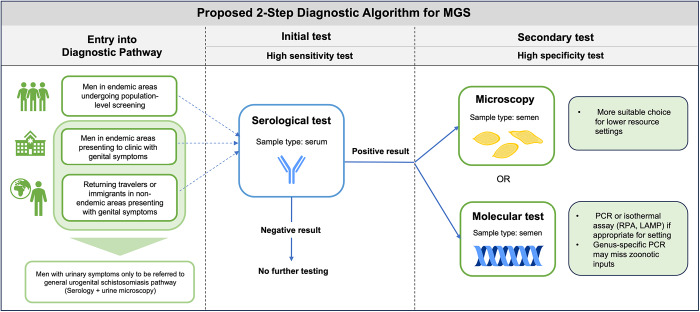

## Introduction

Urogenital schistosomiasis (UGS) is a neglected parasitic disease which affects an estimated 112 million people across sub-Saharan Africa, the Middle East, and more recently Corsica (van der Werf et al., 2003; World Health Organization, 2023). The disease is caused by *Schistosoma haematobium* infection through contact with contaminated freshwater, where larval stages of the parasite, cercariae, penetrate the skin after having been released by their intermediate snail hosts (Ross et al., 2002). Once mature, male and female adult worms pair up and migrate to the venous plexus of the bladder where each pair lays approximately 100–200 eggs per day (Colley et al., 2014). *Schistosoma haematobium* ova are excreted through urine, though roughly half become trapped in tissues (Nation et al., 2020). The presence of the eggs induces a T-helper type 2 (Th2) immune response, leading to chronic inflammation, formation of granulomas and fibrosis, which are responsible for the clinical symptoms and complications associated with the disease (McManus et al., 2020). Diagnosis of UGS typically involves the detection of parasite ova or genetic material in urine (Le and Hsieh, 2017). The only treatment currently available for schistosomiasis is praziquantel, an anthelmintic drug primarily used in mass drug administration programmes which have traditionally targeted school-aged children, but more recently have been expanded to include neglected populations such as pregnant women and preschool-aged children (World Health Organization, 2022).

**Figure 1.**
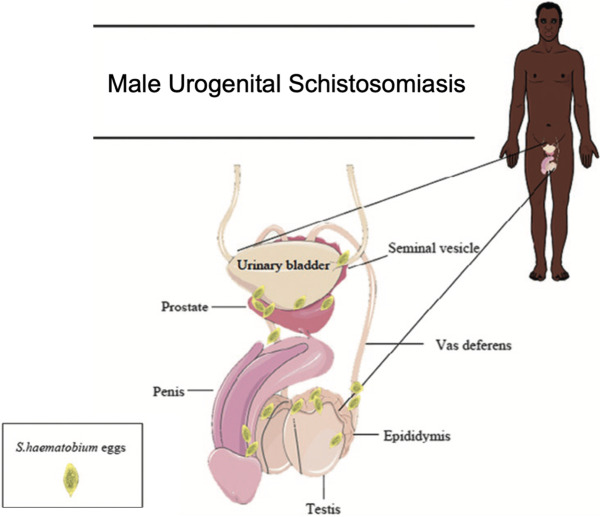
An image depicting the male urogenital organs where *S. haematobium* ova have been found and can lead to male genital schistosomiasis. Adapted image taken from Bustinduy et al. (2022).

Male genital schistosomiasis (MGS) is a distinct gender-specific manifestation of UGS described in males of all ages (Kayuni et al., 2019a; Richter et al., 2025), which occurs when eggs laid by *Schistosoma* species become trapped in the reproductive organs, triggering an immune response and subsequent immunopathology (Figure 1) (Bustinduy et al., 2022). Resultant symptoms include blood in semen (haematospermia), ejaculatory pain, the presence of sperm in urine (spermaturia), pelvic or ejaculatory pain, abnormal ejaculate and inflammation of the testicles and/or prostate (Kayuni et al., 2019a; Bustinduy et al., 2022). Additionally, MGS has been hypothesized to be linked to infertility (Kini et al., 2009; Abdel-Naser et al., 2019), prostate cancer (Figueiredo et al., 2015; Choto et al., 2020a, 2020b), and HIV transmission to female partners, likely through increased viral shedding in seminal fluid (Leutscher et al., 2000a; Midzi et al., 2017; Kayuni et al., 2023a). Diagnosis is performed through the detection of *S. haematobium* ova in genital fluid and/or organs, most commonly by semen microscopy (Kayuni et al., 2019a, 2024b). Although *S. haematobium* is considered to be predominantly responsible for genital schistosomiasis, other *Schistosoma* species have been observed to be involved in MGS in limited case reports (usually in biopsy or pathology studies), including *S. mansoni, S. japonicum* and *S. intercalatum* (Corachan et al., 1994; Lopes et al., 2007; Yu et al., 2013). Recently, *S. mattheei*, a primarily livestock-infecting species though capable of zoonosis, has been implicated in MGS in Malawi (along with a possible *S. haematobium*–*S. mattheei* hybrid) leading to raised concerns about potential zoonotic species involvement in genital schistosomiasis (Kayuni et al., 2024a).

More broadly, surprisingly little is known about the natural history of MGS due to a lack of longitudinal studies. There are no current and accurate estimates of the burden of MGS, with limited case reports and studies conducted in few countries (Bustinduy et al., 2022). Additionally, there is a lack of data on the pathological consequences of MGS if left undiagnosed and untreated. Chronic inflammation as a result of *Schistosoma* egg deposition in the genital area may have similar consequences to sexually transmitted infections such as *Chlamydia trachomatis*, which has been linked to infertility in men (Fode et al., 2016; Keikha et al., 2023). Despite female genital schistosomiasis (FGS) being a similarly neglected disease on a global scale, the relationship between FGS and HIV has resulted in a rise in the number of studies in the area and innovation in diagnostics, including molecular isothermal assays (Archer et al., 2022; van Bergen et al., 2024), computer-assisted image analysis (Holmen et al., 2015; Arenholt et al., 2022), and validation of new sample types (i.e. self-collected swabs) (Sturt et al., 2020). In contrast, MGS remains underreported and understudied. Diagnostic challenges, coupled with a lack of awareness in healthcare systems, contribute to the scarcity of data and the knowledge gap regarding the global burden of MGS (Kayuni et al., 2019a, 2024b; Bustinduy et al., 2022).

A variety of diagnostic tools exist for UGS, including microscopy, antigen detection, antibody detection and nucleic acid amplification tests (NAATs). Accurate MGS diagnosis can be challenging, as traditional diagnostic methods for UGS are often insufficient as ova may be present in semen but absent in urine and vice versa, and a positive antigen or antibody test does not necessarily confirm genital involvement (Kayuni et al., 2019a). Although urine microscopy has occasionally been used as a proxy for MGS diagnosis, previous studies have observed a range of between 27% and 67% of men with MGS have no detectable ova in urine (Leutscher et al., 2000a; van Delft et al., 2007; Kayuni et al., 2021; Makene et al., 2024). Additionally, challenges with sample collection and processing exist, which may influence the performance of diagnostic tests including diurnal variation in ova excretion in seminal fluid, the period of abstinence prior to specimen collection and the method of collection itself (Le and Hsieh, 2017; World Health Organization, 2021). While barriers such as these are overcome in urine samples with repeat sampling (Le and Hsieh, 2017), this poses challenges with semen due to cultural sensitivities and beliefs in some regions surrounding the process of semen collection and processing (Kayuni et al., 2019a). Few studies in only two countries (Madagascar and Malawi) have evaluated the performance of diagnostic tests for MGS or conducted optimization of existing *S. haematobium* diagnostic assays for use with seminal fluid (Leutscher et al., 2008; Kayuni et al., 2021, 2023b). Evaluation of diagnostic tests for MGS is challenging due to the lack of a consensus on a ‘gold standard’ test, and conclusions on test performance are difficult to make, given the absence of laboratory studies on test performance in a controlled setting prior to deployment in the field. Together, these factors have contributed to a gap in the current diagnostic landscape for schistosomiasis, as MGS lags behind.

In comparison to general UGS or FGS, MGS diagnostic development and validation has remained relatively stagnant, resulting in a lack of a clearly defined optimal diagnostic pipeline. Challenges with diagnosis and the lack of accessible, effective diagnostic tests have led to the underreporting of the disease and a knowledge gap regarding its global burden. The aim of this review is to describe the current diagnostic landscape for MGS and applications of available diagnostic tests, discuss ongoing challenges and identify needs and directions for future research in order to propose a roadmap for potential MGS diagnostic algorithms.

## Diagnostic landscape

The diagnostic landscape for MGS is sparse, with only a handful of studies reporting on test performance using quantitative measures of validity (Table 1). Of these, there is substantive heterogeneity across the choice of index and reference tests and limited sample sizes making it difficult to draw overall conclusions regarding the relative performance of different tests.

**Table 1.** Studies reporting measures of validity of diagnostic tests for male genital schistosomiasis

Study	Country	Index test	Reference test	Se (%)	Sp (%)	PPV (%)	NPV (%)
Kayuni et al. (2023b)	Malawi	Semen microscopy (centrifugation)	*Schistosoma* ITS − 2 PCR on semen	14.3	91.5	NR	NR
		Urine microscopy (filtration)	*Schistosoma* ITS − 2 PCR on semen	33.3	93.2	NR	NR
		POC-CCA	*Schistosoma* ITS − 2 PCR on semen	52.4	67.9	NR	NR
Kayuni et al. (2021)	Malawi	Semen microscopy (simple bag method)	Semen microscopy (sedimentation centrifugation)	55.6	97.1	NR	NR
Leutscher et al. (2008)	Madagascar	ECP in semen (in-house ELISA)	Semen microscopy	97	25	52	NR
		SEA in semen (in-house sandwich ELISA)	Semen microscopy	41	93	83	NR
		CAA in serum (in-house sandwich ELISA)	Semen microscopy	76	98	97	NR
		Urine microscopy (filtration)	Semen microscopy	42	83	82	NR

Se = Sensitivity, Sp = Specificity, PPV = Positive Predictive Value, NPV = Negative Predictive Value, POC-CCA = point-of-care circulating cathodic antigen, ECP = eosinophile cationic protein, SEA = soluble egg antigen, CAA = circulating anodic antigen.

The MGS diagnostic landscape can be divided into two different categories: tests for infection, and methods to assess morbidity or chronic disease. Tests for infection can confirm the presence of *Schistosoma* adult worm pairs or their genetic material, and morbidity-related diagnostics assess the pathology or morbidity associated with MGS or *S. haematobium* infection. While these morbidity-related tests are valuable for understanding the broader history and clinical impact of MGS, they are generally insufficient for definitive diagnosis of genital schistosomiasis as any observed morbidity may not be specific to MGS, though they may play a role in aiding the understanding of the natural history of MGS.

### Tests for infection

#### Microscopy

Visualization of *Schistosoma* ova in semen via microscopy is the most commonly used diagnostic method for MGS and can provide quantitative data on excreted egg burden (Kayuni et al., 2019a; Bustinduy et al., 2022), although the sensitivity of this technique is subject to a vast array of factors, including sampling techniques, sample processing, background disease prevalence and infection intensity. Substantial variation has been observed in ova excretion in seminal fluid collected from the same participants across different days, suggesting the diurnal variability and day-to-day variation in ova excretion commonly observed in urine samples is also present in semen (Leutscher et al., 2008). While this barrier is overcome in urine samples with repeat sampling (Le and Hsieh, 2017), this poses challenges with semen due to cultural sensitivities, beliefs and stigma surrounding the process of semen collection and processing (Kayuni et al., 2019a).

The lack of standardization in sample collection poses another challenge in conducting semen microscopy. Considerations which may influence diagnostic performance include the timeframe between sample collection and analysis, ejaculate collection method and period of abstinence prior to sampling. Ejaculation collection method may impact the quality of the semen sample, as coitus interruptus may result in an incomplete sample (World Health Organization, 2021). The WHO laboratory manual for the examination and processing of human semen, which focuses on infertility investigation, provides a template for an analysis report form detailing parameters such as the period of abstinence prior to ejaculation, time between collection and analysis and sample volume (World Health Organization, 2021). Examinations of semen for MGS diagnosis should be accompanied by a similar form. Microscopy may also be used to examine other components in semen, including leukocytes or red blood cells, which may be associated with MGS and have clinical relevance (Leutscher et al., 2005; Long and Kenworthy, 2022).

Methods for conducting microscopy on seminal fluid vary and involve a range of sample preparation techniques, as there is currently no standardized protocol for semen microscopy. Sample concentration methods prior to conducting microscopy may impact sensitivity, as is the case with urine microscopy for detection of *S. haematobium* ova. Filtration has been shown to be more sensitive than centrifugation–sedimentation and is therefore considered the gold standard for urine microscopy (Shaker et al., 1994; Le and Hsieh, 2017). In the few prospective studies that have conducted semen microscopy for MGS, varying methods have been employed including filtration (Leutscher et al., 2008), centrifugation sedimentation (Kayuni et al., 2021, 2024a), and direct microscopy, including visualising ova within a transparent collection bag, often with no sample concentration or pooling (Kayuni et al., 2019b, 2021; Makene et al., 2024). However, no studies have compared the performance of these methods in seminal fluid, which may be due to challenges with low sample volume. Further work is needed regarding laboratory-based evaluation of semen processing methods using simulated samples or samples spiked with *S. haematobium* ova in efforts to develop a standardized microscopy protocol for semen microscopy, including the standardized reporting of results (i.e. eggs/mL).

#### Antigen detection

Various antigens produced and secreted by *Schistosoma* species into the host bloodstream can be detected and used in the diagnosis of MGS, although antigen detection in urine or sera alone is insufficient to indicate genital involvement (Kayuni et al., 2019b). Circulating anodic antigen (CAA) and circulating cathodic antigen (CCA) are regurgitated by live worms and can be detected in urine and serum as a highly sensitive method of identifying active infection and assessing infection intensity (van Lieshout et al., 2000). CCA can be detected via a commercially available, point-of-care device (POC-CCA). Although the POC-CCA assay presents an inexpensive, field-applicable option for intestinal schistosomiasis diagnosis, its use in MGS diagnosis is limited due to its inability to reliably detect *S. haematobium* (Kayuni et al., 2019b). Its applicability in MGS studies to date has consisted of aiding identification of coinfected patients, or those with both *S. haematobium* and *S. mansoni* (Kayuni et al., 2019b). Alternatively, CAA is measured through the upconverting reporter particle-lateral flow assay (UCP-LF CAA) developed by Leiden University Medical Center, which has been evaluated in a variety of settings and has exhibited high sensitivity and specificity across all species of human-infecting schistosomes (Corstjens et al., 2020). Although not yet available in a point-of-care format, work on the development of a rapid test is ongoing with the aim of producing a commercially available test capable of detecting CAA from finger prick blood (FIND, n.d.).

CAA has had limited applications in MGS diagnosis to date, as the presence of CAA can only confirm active *Schistosoma* infection, not genital disease. However, it has been used in previous MGS studies in conjunction with other diagnostic methods, and CAA in serum has been found to be a strong predictor of ova excretion in semen at the population level (Leutscher et al., 2008). The UCP-LF CAA assay was recently used with seminal plasma (rather than serum or urine) for the first time, as part of a longitudinal cohort study of fishermen in Malawi (Kayuni et al., 2019b). Although capable of detecting low levels in some samples (1 pg/mL), few samples had sufficient volume and most were unable to be successfully analysed (Kayuni et al., 2019b). The applicability of CAA in the MGS diagnostic landscape remains questionable, as several challenges exist. As CAA is released into the host bloodstream by adult worms and excreted in urine, both serum and urine are useful sample types for its detection, whereas the level of CAA in semen remains unknown and its significance unclear. Any CAA detected in semen cannot be ruled out as cross-contamination with urine where CAA is present in higher levels (van Lieshout, L. Personal communication). Additionally, seminal fluid is a relatively low-volume sample, which may be inadequate for certain steps in the UCP-LF CAA protocol. Further investigation is warranted into detection of antigens which may be more suitable for use in MGS diagnosis, such as egg-derived circulating antigens.

#### Antibody detection

Serological testing for anti-*Schistosoma* antibodies is a highly sensitive diagnostic method that is often used in areas of low endemicity to ascertain exposure (Hinz et al., 2017). Antibodies to a variety of antigens can be detected including cercarial antigen, adult worm antigen or egg antigens (Hinz et al., 2017). The 2022 WHO guideline on the control and elimination of human schistosomiasis emphasized the importance of utilizing serological tools as transmission declines, due to their sensitivity and ability to detect low-intensity infections (World Health Organization, 2022). Potential limitations include cross-reactivity with other helminths, infrastructure and equipment requirements and the inability to distinguish between active and past infections (Hinz et al., 2017). Of the current commercially available serological tests, many use antigens derived from *S. mansoni* which may impact diagnostic performance when used to detect antibodies to *S. haematobium* (Hinz et al., 2017). Additionally, serological testing is unable to diagnose MGS specifically as a positive serological test result does not indicate genital involvement. The role of serological testing in the MGS diagnostic landscape is largely undefined, as usage has previously been limited to returning travellers as an initial screening tool prior to specific diagnosis of MGS (Lang et al., 2017; Roure et al., 2024; Warrell et al., 2024).

Serological tests have the potential to be powerful screening tools in areas where the prevalence of MGS is relatively low and may serve as a primary screening tool for case identification. The WHO guideline suggests that a two-step diagnostic approach may be warranted as certain areas approach elimination of schistosomiasis. This would consist of an initial test with high sensitivity such as serology, followed by a second confirmatory test with high specificity (World Health Organization, 2022). While some studies have already proposed potential algorithms that meet these guidelines for standard forms of schistosomiasis (intestinal and urogenital) (Mesquita et al., 2022), none have investigated diagnostic algorithms for MGS. Serological testing may provide a useful first step in a diagnostic algorithm for MGS in low prevalence areas in order to focus more specific and costly testing on those who are most likely to be at risk.

#### Nucleic acid amplification tests

Nucleic acid amplification tests (NAATs) can be used in urogenital schistosomiasis diagnosis to overcome issues with sensitivity and specificity and facilitate detection of low-intensity infections (Le and Hsieh, 2017). The use of molecular assays may alleviate other diagnostic barriers such as the diurnal excretion of ova and allow for the detection of infections that may otherwise be missed via microscopy due to low numbers of ova (low-intensity infections), however the high costs and equipment/infrastructure requirements associated with most molecular assays pose a barrier to their usage in resource-constrained settings (Le and Hsieh, 2017; Keller et al., 2020).

Molecular diagnostics have only recently been utilized for MGS diagnosis, with only two studies reporting the use of NAATs to detect *Schistosoma* DNA (internal transcribed spacer [ITS2] region) most likely from ova themselves, in semen (Kayuni et al., 2023b, 2024a). Kayuni *et al*. (2023) pioneered the use of real-time PCR in MGS diagnosis and found its usage alongside microscopy increased the number of men found to have MGS, indicative of its high sensitivity (Kayuni et al., 2023b). Interestingly, the study observed little concordance between semen microscopy and PCR results, finding that 60% (6/10) of cases who were positive via microscopy were not PCR positive (Kayuni et al., 2023b). The authors hypothesize that this discordance could be due to ‘old’ infections and the presence of calcified eggs in genital tissue which are released in seminal fluid and can be detected via microscopy but not by DNA-based methods (Kayuni et al., 2023b). The method of DNA extraction could have also contributed to this finding, as optimal nucleic acid extraction methods from semen remain unknown. Further investigation is needed into the performance of molecular techniques versus microscopy, including a comprehensive diagnostic evaluation of both methods using quantitative measures of validity while taking into account the lack of current suitable ‘gold’ standard. Miracidial hatching could be attempted on ova recovered from semen when conducting microscopy, in order to confirm the existence of an active infection with viable ova that should also be able to be detected by PCR.

Isothermal assays such as loop-mediated isothermal amplification (LAMP) and recombinase polymerase amplification (RPA) have been used in general schistosomiasis diagnostics and have recently been validated for the detection of *S. haematobium* from cervicovaginal swabs and subsequent diagnosis of FGS, however have not yet undergone evaluation for use with seminal fluid (Archer et al., 2022; van Bergen et al., 2024). Isothermal diagnostic tools may present a field-applicable alternative to PCR and can help alleviate barriers to molecular testing in resource-constrained settings as they are often rapid, portable, and may not require a reliable cold chain or power source (Bustinduy et al., 2022). There is a need for optimisation and validation of these tests for use with semen in order to increase accessibility of MGS diagnosis in field or point-of-care settings.

### Morbidity assessment

#### Symptom questionnaires

Questionnaires and surveys have been extensively validated for community screening and are often employed in large multicountry studies in order to assess urogenital schistosomiasis in a simple, rapid and inexpensive manner (Lengeler et al., 2002a, 2002b; Le and Hsieh, 2017). Individual-level questionnaires may suffer from lower sensitivity than at the community level, particularly with light intensity infections due to variability in symptom reporting and the nonspecific nature of some symptoms, but are still an accessible and low-cost means of diagnosing individuals (Lengeler et al., 2002a). While questionnaires for urogenital schistosomiasis focus on haematuria as the main diagnostic indicator, the few studies which have utilized surveys on MGS symptoms tend to encompass a wide variety of genitourinary symptoms. However, questionnaires on MGS have so far been limited to relatively narrow research settings in few countries (Kayuni et al., 2021; Makene et al., 2024; Roure et al., 2024) and no large-scale surveys have been conducted. These questionnaires include symptoms such as haematospermia, dyspareunia or painful ejaculation, which are indicative of genitourinary involvement but are not pathognomonic for MGS (Roure et al., 2024). In this context, as these symptoms can also be caused by sexually transmitted infections, it is important for studies to utilize questionnaires alongside other diagnostic tools such as microscopy, reagent strips, molecular assays or serological testing in order to confirm diagnosis (Lengeler et al., 2002b; Roure et al., 2024).

#### Ultrasonography

**Figure 2.**
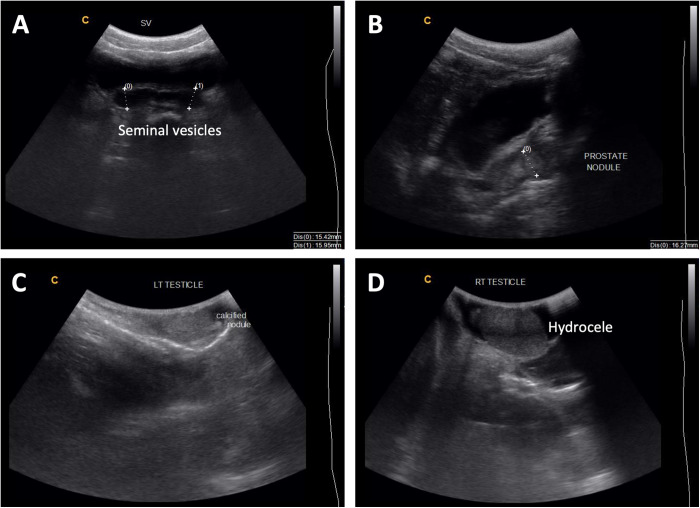
(A) Enlarged seminal vesicles (more than 15 mm normal size) with calcification of walls; (B) 16 mm nodule in the prostate and thickened urinary bladder walls; (C) calcified nodule in the left testis; (D) hydrocele of the right testis. Photo credit: S. Kayuni (Kayuni et al., 2022).

Ultrasonography is a valuable imaging modality for assessing MGS-related morbidity, particularly for identifying structural abnormalities and calcifications associated with chronic infections (Kayuni et al., 2019a; Richter et al., 2025). Transrectal, transabdominal and trans-scrotal ultrasonography can be utilized to visualize pathologies related to MGS, including nodules or calcification of the testes, hydrocele, enlarged or asymmetrical seminal vesicles and prostate masses, calcification, and hyperechogenicity (Figure 2) (Vilana et al., 1997; Richter, 2000; Ramarakoto et al., 2008; Kayuni et al., 2022). When conducting ultrasonography of the bladder for general urogenital schistosomiasis, images can be assessed according to standardized guidelines (i.e. Niamey protocol) allowing for consistency across different studies and populations, though no such guidelines are specifically available for genital pathology (Richter et al., 2000). Although abnormalities observed via ultrasonography may not be specific to schistosomiasis, some characteristic schistosomiasis-related pathologies such as nodules or calcification in the genital area (i.e. prostate, testes, seminal vesicles) can provide important indicators when interpreted alongside clinical history and other diagnostic tools used to confirm MGS (Kayuni et al., 2022; Richter et al., 2025). Portable ultrasonography is suitable for resource-constrained or field settings and can also serve as a tool for monitoring treatment efficacy, particularly by assessing the resolution of lesions or reduction in morbidity following the administration of praziquantel (Kayuni et al., 2022).

#### Other biomarkers

Several known biomarkers of urinary schistosomiasis have been explored in MGS as markers of infection or morbidity. Soluble egg antigen (SEA), an antigen secreted by *Schistosoma* eggs, have been investigated as a potential biomarker for MGS with a positive correlation observed between seminal SEA and egg counts (Leutscher et al., 2008). Similarly, eosinophil cationic protein (ECP), which is released from eosinophil granules and has cytotoxic effects against schistosomula (Ackerman et al., 1985), has been examined as a potential biomarker for MGS due to its established role in urinary schistosomiasis in monitoring inflammation (Reimert et al., 2000; Leutscher et al., 2000b, 2008; Midzi et al., 2003). Men with *S. haematobium* ova in semen have been observed to have higher semen ECP levels compared to those without; however, no difference in overall ECP positivity has been observed between the two groups, nor any correlation with ova count (Leutscher et al., 2008). SEA and ECP in seminal fluid may therefore be useful as additional biomarkers for MGS in terms of morbidity, but are not suitable alone in terms of clinical diagnosis of MGS (Leutscher et al., 2008).

## Future directions

**Figure 3.**
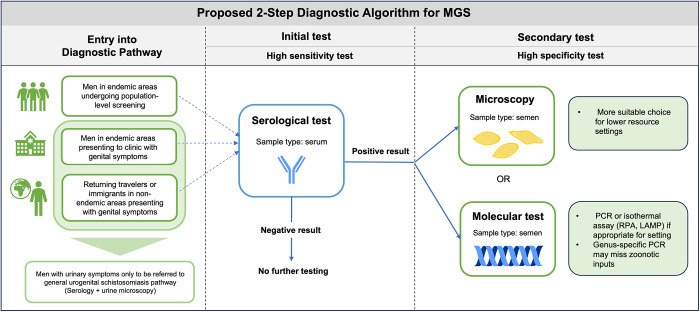
Proposed potential diagnostic algorithm for MGS.

The diagnostic landscape for MGS faces a multitude of challenges and requires further efforts to improve performance and reduce knowledge gaps at all levels: sample collection, processing, storage and analysis. With no current diagnostic guidance for MGS, we propose a potential diagnostic algorithm for MGS in line with recent WHO guidelines consisting of an initial high-sensitivity serological test, followed by a high specificity secondary test (semen microscopy or molecular-based test) (Figure 3). This algorithm could be adapted based on endemic setting or population (i.e. age group) and tests could be selected for the appropriate resource level, i.e. microscopy used as the high specificity test in more resource-constrained settings where molecular diagnostics may not be accessible. It could be altered based on background prevalence and endemicity, for example while serological tests may be more appropriate for lower prevalence settings, alternatives could be considered for highly endemic settings where serology may not be as suitable, such as detection of CAA in serum (particularly with the current efforts to develop a point-of-care rapid test). In order to ensure optimal performance of such an algorithm, further investigation into sample collection and processing is required and standardization of methods is crucial.

Sample collection presents an initial hurdle to diagnostic evaluation for MGS given the wide range of techniques used and lack of comparison between them. Standardization of seminal fluid collection methods are needed, along with investigation into the impact of factors such as period of abstinence prior to collection, collection method (i.e. masturbation or coitus interruptus), and time of day of sampling as it is unknown how these influence ova excretion in semen and diagnostic sensitivity. This will be important to aid in the development of a standardized protocol for semen microscopy for the detection of *Schistosoma* ova, in a similar manner to the WHO protocol developed for investigation of male infertility (World Health Organization, 2021). Further work is necessary to better understand cultural perceptions and hesitancy surrounding the collection of semen samples with regards to MGS diagnosis, in a similar manner to previous studies on HIV or Ebola (Price et al., 2005; Kutalek et al., 2020). There is also a need to integrate these considerations into the diagnostic approach. Community engagement should be prioritized to foster acceptability of sampling and identify potential barriers to sample collection (Kayuni et al., 2019a).

Additional optimization of existing molecular diagnostic tests for *S. haematobium* may be required for use with semen. Seminal fluid contains a large amount of DNA and has been shown to contain inhibitors, which may affect amplification, although the mechanisms of potential inhibition are unknown (Peeling and Embree, 2005). When applying existing molecular diagnostic tools to MGS diagnosis, care should be taken to first evaluate test performance with semen to ensure sensitivity is not compromised. Optimal nucleic acid extraction methods from semen should be investigated, including strategies that have been shown to increase extraction efficiency from *Schistosoma* ova in other sample types (i.e. bead beating) (Pomari et al., 2019).

Though sensitive nucleic acid-based tests are a promising way forward for the diagnosis of MGS, challenges persist with sample storage transport, particularly in resource-constrained or field settings. Molecular methods, such as PCR, require a reliable cold chain which may not be available in many areas where urogenital schistosomiasis is highly endemic, leading to concerns about sample stability and quality when samples are stored in sub-optimal conditions for extended periods of time. Future studies should explore how varying sample storage and processing conditions affect DNA recovery from seminal fluid.

There is a need for isothermal assays to be evaluated for the detection of *S. haematobium* from semen and subsequent diagnosis of MGS in order to alleviate barriers to DNA-based testing in resource-constrained settings. The *S. haematobium* RPA (Sh-RPA) assay presents a promising opportunity for increasing the accessibility of molecular testing in field settings, as it is portable, rapid, and uses shelf stable reagents which negates the need for reliable cold chain (Rostron et al., 2019). It has shown to be highly sensitive (93.3%) and specific (96.6%) for the detection of *S. haematobium* in genital self-swabs for diagnosis of FGS, though has not yet been evaluated with semen or utilized for MGS diagnosis (Archer et al., 2022). Although the Sh-RPA has been estimated to be more costly per sample compared to real-time PCR ($4 USD vs. $1.5 USD), the cost of the portable RPA device is less (between $5,000 and 10,000 USD) than that of a real-time PCR thermocycler ($10,000 and 30,000 USD) (Archer et al., 2022). The ribosomal intergenic spacer (IGS)-Sh-LAMP is another isothermal diagnostic assay, which has shown promise in FGS diagnosis, and has recently been developed into an instrument-free method using low cost 3D-printed materials, though has only been evaluated with a small number of samples (van Bergen et al., 2024). Further validation of this assay is warranted with well-characterised samples on a larger scale, alongside optimization for use with seminal fluid.

With the recognition of zoonotic and *Schistosoma* hybrid species as a potential public health threat, new diagnostic tools are urgently needed (Leger and Webster, 2017). Preliminary evidence suggests these species contribute to the burden of MGS, with Kayuni and colleagues observing both *S. haematobium* and *S. mattheei* ova in the semen of a man with MGS in Malawi, and another with possible *S. haematobium–mattheei* hybrid DNA (Kayuni et al., 2024a). A substantial knowledge gap remains regarding the role of *S. haematobium–bovis* hybrids in the burden of MGS, which are common in West African regions (Stothard et al., 2020). Traditional diagnostic tools such as microscopy are insufficient for surveillance of hybrid species as ova may not be morphologically distinct (Stothard et al., 2020). The use of molecular assays capable of detecting zoonotic and hybrid species such as the novel high-resolution melt (HRM) real-time PCR assay developed by Cunningham et al. (2024) should be prioritized in areas and populations where there is high potential for zoonotic infection (Cunningham et al., 2024; Kayuni et al., 2024a). Evaluation of current diagnostic tools with samples containing zoonotic and hybrid ova and/or genetic material are necessary to understand the impact of hybrid infections on diagnostic performance and also their role in ectopic ova excretion or elevated pathology.

The lack of a suitable reference standard for MGS diagnosis has directly contributed to the knowledge gap on burden across sub-Saharan Africa. New diagnostic tools, particularly field-applicable molecular assays including those capable of identifying zoonotic or hybrid species, are urgently needed. Comprehensive diagnostic evaluation studies are essential to assess the performance of current diagnostic tools considering the lack of suitable ‘gold standard’. Here, we have proposed a potential diagnostic algorithm for MGS in accordance with WHO guidelines, which can be adapted based on local context. Future work on the development of standardized protocols for sample collection, processing and analysis will maximize sensitivity and help streamline the diagnostic pipeline. The importance of community sensitization and engagement cannot be understated, as barriers persist with hesitancy of semen sample collection. The diagnostic gap for MGS presents a threat to the goal of elimination of schistosomiasis as a public health problem as without sensitive and accessible tests, the burden will continue to be underreported and impacts to the sexual and reproductive health of men will persist. Addressing the development and comprehensive evaluation of diagnostic tools for MGS is an urgent priority to overcome decades of neglect, advance effective disease management, and achieve equitable global health outcomes.
